# Cardiac surgery for patients with schizophrenia: clinical experience of six patients

**DOI:** 10.1007/s00595-021-02369-4

**Published:** 2021-09-04

**Authors:** Mutsuo Tanaka, Minoru Okamoto, Kensho Yamashita

**Affiliations:** 1 Department of Cardiovascular Surgery, National Hospital Organization Kumamoto Medical Center, 1-5 Ninomaru, Chuou-ku, Kumamoto, 860-0008 Japan; 2 Department of Psychiatry, National Hospital Organization Kumamoto Medical Center, 1-5 Ninomaru, Chuou-ku, Kumamoto, 860-0008 Japan

**Keywords:** Cardiovascular surgery, Schizophrenia, Mental illness

## Abstract

**Purpose:**

The incidence of schizophrenia in Japan is 0.7%, which is similar to the worldwide incidence. The mortality rate of patients with schizophrenia is reported to be higher than that of the general population, and cardiovascular disease is high among the causes of death. Hence, strategies for cardiovascular surgery for patients with schizophrenia are necessary.

**Methods:**

We studied six patients with schizophrenia (five males, one female) who underwent cardiac surgery in our hospital between April 2008 and December 2019.

**Result:**

The mean age was 63.6 years. The surgical procedures were coronary artery bypass grafting (CABG) (*n* = 4), CABG concomitant with valve procedures (*n* = 1), and resection of myxoma (*n* = 1). There were no major cardiovascular complications and no other fatal complications. The mean observation period was 1510.6 ± 1430.1 (140–4068) days, the mean post-operative hospital stay was 17.8 ± 3.5 (13–22) days, and there was no mortality within 30 days after surgery.

During the observation period, one patient died. The survival rate was 83.3% at 1, 3, and 5 years.

**Conclusion:**

Cardiac surgery for patients with schizophrenia is possible with careful monitoring of indications and perioperative management.

## Introduction

According to a report by the Japanese Ministry of Health, Labor and Welfare in 2018, more than 4 million patients were being treated for mental illness, and 704,000 were dementia patients [1]. Dementia has become a global social problem in recent years [2]. On the other hand, the number of patients with schizophrenia in Japan was 793,000 [1]. In addition, the total number of inpatients with mental illness was 302,000, with schizophrenia accounting for 154,000 of these cases [1]. The prevalence of schizophrenia in the Japanese entire population was 0.04–1.1% [1, 3], which is similar to the prevalence worldwide [4].

Our institution offers psychiatric emergency services in addition to general psychiatric treatment. Hence, patients with mental illness complicated with cardiovascular disease (CVD) are encountered. In this report, the treatment progress of patients with schizophrenia who underwent cardiac surgery was evaluated retrospectively, and we report the results with a review of the relevant literature.

## Methods

### Data source

The survey was conducted retrospectively using hospital records of patients managed between April 2008 and December 2020.

### Patients

Patients with schizophrenia who underwent cardiac surgery in our hospital were selected. The inclusion criteria were a diagnosis of schizophrenia that met the F20 criteria in the International Classification of Disease-10 or the 295 classifications of the Diagnostic and Statistical Manual of Mental Disorders-II, and regular treatment before surgery.

### Clinical outcomes

We assessed the patient characteristics, surgical procedures, post-operative mortality, incidence of each complication, and survival time.

### Ethical approval

This report was approved by our institutional ethics committee.

## Results

During the observation period, a total of 14 patients with schizophrenia underwent cardiovascular surgery, including surgery for peripheral blood vessels. Among the 14 patients, six underwent cardiac surgery and were surveyed.

### Patient characteristics

The study population included five males and one female. The mean age was 63.6 ± 9.4 years (48–76 years). The diseases included ischemic heart disease (*n* = 4), aortic stenosis (*n* = 1), and myxoma in the left atrium (*n* = 1). The mean body surface area was 1.64 ± 0.19 (1.4–1.85) m^2^, and the mean body mass index (BMI) was 24.3 ± 5.1 (19.1–33.2) kg/m^2^. One patient was staying at a psychiatric hospital, while five patients lived at home (single living *n* = 4, family living together *n* = 1).

The risk factors included hypertension (*n* = 3), diabetes mellitus (DM) (*n* = 1), hyperlipidemia (*n* = 1), mean serum creatinine 0.89 ± 0.21 (0.8–1.18) mg/dl, a past history of cerebral disorder (*n* = 3), CVD (*n* = 3), respiratory dysfunction (history of chronic obstructive pulmonary disease or percent predicted forced expiratory volume in one second < 70%) (*n* = 2), smoking (*n* = 4), mean New York Heart Association classification 2 ± 1.09 (1–4), and mean ejection fraction of left ventricle 53.1 ± 15.7 (34.0–74.0%). The mean period of schizophrenia treatment was 25 ± 9.96 (15–42) years. Two or more antipsychotics were administered in all cases, and benzodiazepine was administered in combination in five cases. The mean dosage of a chlorpromazine (CP)-equivalent antipsychotic drug was 679.5 ± 744.2 (15–1812) mg [Table 1].

**Table 1:** Preoperative patient characteristics

Characteristics of all shizophrenic patients who underwent cardiovascular surgery	Number
Total shizophrenic patients	14
Cardiac surgery	6
Thoracic aortic aneurysm	1
Abdominal aortic aneurysm	6
Self-harm of blood access for hemodialysis	1
Characteristics of shizophrenic patients who underwent cardiac surgery	Number
Preoperative diagnosis	
Ischemic heart disease	4
Aortic stenosis	1
Myxoma	1
Sex	
Male	5
Female	1
Age (years old) mean ± SD^1)^ ( range)	63.6 ± 9.4 (48–76)
Body Surface Area (m^2^) mean ± SD^1)^ (range)	1.64 ± 0.19 (1.4–1.85)
Body mass index (Kg/m^2^) mean ± SD^1)^ (range)	24.3 ± 5.1 (19.1–33.2)
Daily living situation	
Hospital or facility stay	1
Home (living alone)	5 (4)
Risk factors	
Hypertension	3
Diabetes Mellitus	1
Hyperlipidemia	1
Hemodialysis	0
Cerebral vascular disease	1
Cardiovascular disease	3
Respiratory disorder (Chronic obstructive pulmonary disease or %FEV1.0^2)^ < 70%)	2
Smoking	4
Chronic kidney disease	0
Serum Creatine (mg/dl) mean ± SD^1)^ (range)	0.89 ± 0.21 (0.8–1.18)
Classification of New York Heart Association mean ± SD^1)^ ( range)	2 ± 1.09 (1–4)
Ejection fraction (%) mean ± SD^1)^ ( range)	53.1 ± 15.7 (34.0–74.0)
Schizophrenia treatment status	
Treatment period (years) mean ± SD^1)^ (range)	25 ± 9.96 (15–42)
Administration of two or more antipsychotic drugs	6
Combined administration with benzodiazepine	5
Chlorpromazine equivalents (mg) mean ± SD^1)^ (range)	679.5 ± 744.2 (15–1812)

^1)^*SD* standard deviation

^2)^*%FEV1.0* percent predicted forced expiratory volume in one second

### Surgical procedures

The surgical procedures were CABG on-pump beating (*n* = 3); CABG concomitant with valve procedures (*n* = 2); mitral valve replacement with bioprosthesis, tricuspid annuloplasty, and left appendage closure (*n* = 1); aortic valve replacement with bioprosthesis (*n* = 1); and resection of myxoma (*n* = 1). The mean surgical time was 354.2 ± 149.8 (200–643) minutes, the mean cardiopulmonary bypass time was 184.8 ± 117.2 (109–418) minutes, and the mean aortic clamping time (excluding CABG) was 141.3 ± 106.2 (39–251) minutes. There were no patients who required re-operation; however, one patient was admitted for emergency treatment. Four patients received transfusion [Table 2].

**Table 2:** Surgical procedures

Characteristics	number
Procedure	
CABG^1)^ on-pump beating	3
CABG^1)^ concomitant with valve procedure Mitral valve replacement with bioprosthesis Tricuspid annular plasty Left appendage closure	1
Aortic valve replacement with bioprosthesis	1
Resection of myxoma	1
Operation time (min)mean ± SD^2)^ ( range)	354.2 ± 149.8 (200–643)
CPB^3)^ time (min) mean ± SD^2)^ ( range)	184.8 ± 117.2 (109–418)
Aorta clamping time (min) mean ± SD^2)^ ( range) (Excluding the three cases of CABG^1)^ on-pump beating)	141.3 ± 106.2 (39–251)
Blood transfusion	4
Re-operation	0
Emergent / Urgent	1

^1)^*CABG* coronary artery bypass grafting

^2)^*SD* standard deviation

^3)^*CPB* cardiopulmonary bypass

Postoperative course.

The mean observation period was 1510.6 ± 1430.1 (140–4068) days, the mean post-operative hospital stay was 17.8 ± 3.5 (13–22) days, and there was no mortality (within 30 days after surgery). The post-operative complications included paroxysmal atrial fibrillation (*n* = 1) and delirium (*n* = 1); however, no fatal complications occurred [Table 3].

**Table 3:** The post-operative course

Characteristics	number
Intubation time (hr) mean ± SD^1)^ (range)	12.8 ± 13.6 (3–39)
ICU^2)^ stay (day) mean ± SD ( range)	3.7 ± 2.3 (2–8)
Hospital stay after operation (day) mean ± SD^1)^ (range)	17.8 ± 3.5 (13– 22)
Discharge status	
Home	1
Transfer to other hospital	5
Complications	
MACE^3)^	0
Deep wound infection	0
Prolonged ventilation > 72 h	0
Renal failure ( required hemodialysis)	0
Paroxysmal atrial fibrillation	1
Delirium	1
Observation period (day) mean ± SD^1)^ ( range) Survival (%)	1510.6 ± 1430.1 (140–4068)
1 year	83.3
3 years	83.3
5 years	83.3

^1)^*SD* standard deviation

^2)^*ICU* intensive care unit

^3)^*MACE* major adverse cardiac events

The mean survival rate was 83.3% at 1, 3, and 5 years [Fig. 1]. During the observation period, one patient died of sudden death on the sixth day after pheochromocytoma surgery. Some gastrointestinal complications were suspected on autopsy imaging; however, the cause of death could not be identified, and no pathological autopsy was performed.

## Discussion

There are two important issues regarding the treatment of CVD or cardiovascular surgery complicated with schizophrenia: (1) the patient has a higher risk of CVD, and (2) the patient has a higher risk of perioperative management difficulties.

### Higher risk factor of CVD

The annual mortality rate of patients with schizophrenia is approximately 2–2.5% [5, 6]. Although this includes a high suicide rate, it is approximately 2–4 times higher than the rate of the general population [7]. Sweeting et al. reported a study of 683 patients with schizophrenia who died from CVD, and noted that the main cause of the increased mortality rate was ascribed to coronary artery disease (CAD) [8].

This high cardiovascular mortality rate has been reported to be related to lifestyle, as the rates of DM, metabolic syndrome, and smoking are high [9–11]. In the present study, only one patient had DM; however, other lifestyle factors, single-living (*n* = 4), BMI 24.3 ± 5.1 kg/cm^2^, and smoking (*n* = 4), remained unchanged after treatment.

Furthermore, the content and dosage of antipsychotics significantly affected CVDs other than CAD. In particular, QT prolongation on the electro-cardiogram (ECG) and deep venous thrombosis (DVT)/pulmonary embolism (PE) were observed. The cardiovascular risk of multi-drug treatment with antipsychotics is 2.5-fold that of single-drug treatment [12], and 1.9-fold that of patients who receive concomitant benzodiazepines [13]. These side effects are proportional to the dose, and when converted to a CP-equivalent drug, ECG abnormalities are likely to occur at doses of ≥ 1000 mg, while DVT/PE is likely to occur at doses of ≥ 600 mg [14, 15]. In the present study, all six patients received multi-drug treatment, and five patients received concomitant treatment with benzodiazepines. The average dosage of antipsychotics was 679 mg in the patients who received a CP-equivalent drug (two patients received > 1000 mg and four patients received < 600 mg). However, there were no abnormal ECG or DVT/PE complications.

### Perioperative management difficulties

There have been some reports about surgery for patients with mental diseases, including schizophrenia, at other surgical departments. Although it is difficult to compare other surgeries with cardiac surgery because the invasiveness is different, cardiac surgery for patients with schizophrenia may be possible if they meet certain conditions. To perform perioperative management safely, schizophrenia should be considered in the pre-operative, intra-operative, and post-operative periods.

### Problems in the pre-operative period

Patient selection was an important problem in this period. There were no clear criteria for surgical indications for schizophrenic patients because it is difficult to quantify their mental state. Although this is a difficult issue, it is a similar concept to “frailty,” which is used for elderly patients, as activity, clinical symptoms (such as acute to chronic confusional phases); and cognitive ability vary among patients with schizophrenia. Hence, cardiac surgery may not be suitable for patients with schizophrenia who have reduced activity, reduced cognitive ability, or an acute confusional phase. Cardiac surgeries have not been performed for such patients in our hospital; however, all six patients of this report were able to maintain their activities of daily living without physical support, with the exception of reliable medication management.

### Problems during the operative period

Low blood pressure, especially severe hypotension following general anesthesia, occurred in 5–20% of the patients with schizophrenia. This is due to the side effects of antipsychotics [16], as many antipsychotics are antagonistic to the α1 receptor, which has a vasopressor effect. This effect causes poor norepinephrine, adrenocorticotropic hormone, and cortisol responses during surgery. However, the discontinuation of antipsychotics before surgery to avoid low blood pressure is not recommended because it may exacerbate post-operative delirium [16]. In the present study, most patients presented with hypotension during surgery. One patient was complicated by pheochromocytoma, which may induce catecholamine crisis. This patient had low blood pressure and was frequently treated with catecholamines during surgery.

### Problems in the post-operative period

The onset of delirium is an important and challenging problem for all medical staff. To prevent the onset of delirium, especially in the acute post-operative period, dexmedetomidine hydrochloride has been used as alternative medication from before extubation or starting the oral intake in our hospital. Previous studies administered dexmedetomidine hydrochloride for nighttime sedation concomitant with oral medications during ICU care [17]. If the onset of delirium is suspected, the elimination of its causes, such as pain, is necessary and haloperidol and dexmedetomidine hydrochloride are administered. After starting oral medications, the regimen of antipsychotics that was administered pre-surgery is continued [16]; however, benzodiazepines are stopped because they have been reported to be a risk factor for delirium [18]. If adjustments of antipsychotics are needed due to factors such as overdose, abnormal ECG or DVT/PE complication, or delirium onset, it is better to adjust antipsychotics by consulting psychiatrists, as many patients with schizophrenia have a long medical history (the mean treatment period was 25 years in this report), and cardiovascular surgeons cannot easily make adjustments to psychiatric medication. In addition to medication control, we performed standard management of patients, such as early removal of intravenous or drainage tubes, pain control, and rehabilitation. These measures were successful in all cases except one. The one case was an emergent case with advanced delirium after surgery; however, the patient’s symptoms improved with the cooperation of a psychiatrist and management in a psychiatric ward.

In addition to delirium, lower pain thresholds and difficulty in communication are also important for the treatment of patients with schizophrenia [19].

Regarding lower pain thresholds, although this case was not included in this report, one patient who underwent open abdominal aortic aneurysm surgery developed delirium 2 days after surgery and manually reopened the surgical wound by himself, despite there being no complaint of wound pain at the time.

We experienced one case that involved difficulty in communication. The patient underwent CABG before pheochromocytoma surgery, which successfully resolved, and resection of pheochromocytoma was performed 4 months after CABG; however, the patient died suddenly at 6 days after surgery. This patient had no complaints except for reduced activity and appetite. Li et al. reported that the occurrence of such events was related to surgeons and medical staff having less experience with patients with schizophrenia [19]. Thus, it is desirable to consult psychiatrists before surgery.

Finally, medication management is also an important factor because this affects the surgical treatment plan. It has been reported that approximately one-third of outpatients with schizophrenia are unable to manage their medications properly [20]. In fact, similar cases were found in this report. For example, a valve replacement procedure was performed in two patients (both were 62 years of age) in this report and a bioprosthetic valve was used in both patients due to the risk of mismanagement of anticoagulant therapy. Prior to surgery, a relative of one of the patients told us “I've seen medicine lying on the floor several times”; thus, the patient may not have been able to take the correct medication. This issue may have been related to the lifestyle of the patients, as both patients were living alone and supervision to ensure correct medication usage may not have been sufficient. In the present study, the taking of medication was confirmed under the supervision of nurses, and there were no mistakes, refusal of medications, or complications during hospitalization in any case. In another case, the prothrombin time was not regularly examined after surgery in a patient who was only attending a psychiatric hospital. This psychiatric hospital adjusted the dose of warfarin based on a blood test that was performed in our department every 3 or 6 months.

The results of this report cannot be applied to all patients with schizophrenia, because the number of patients was small and the severity and factors varied among the patients. Furthermore, these findings were not compared with a control group of non-psychiatric patients. Further studies are needed.

## Conclusion

Patients with schizophrenia have risk factors for CVD and high mortality in relation to the disease itself, as well antipsychotic medication. The severity of schizophrenia varied and there were many factors to consider, including the difficulty of perioperative management and device selection. However, cardiac surgery for patients with schizophrenia is possible if the patients have the ability to live independently. The cooperation of psychiatrists is desirable before surgery.

## Abbreviations

CABG: Coronary artery bypass grafting
CVD: Cardiovascular disease
BMI: Body mass index
DM: Diabetes mellitus
CP: Chlorpromazine
CAD: Coronary artery disease
ECG: Electrocardiogram
DVT: Deep venous thrombosis
PE: Pulmonary embolism
